# Genome-Wide Gene Expression Analysis Suggests an Important Role of Hypoxia in the Pathogenesis of Endemic Osteochondropathy Kashin-Beck Disease

**DOI:** 10.1371/journal.pone.0022983

**Published:** 2011-07-29

**Authors:** Feng Zhang, Xiong Guo, Weizhuo Wang, Hua Yan, Chunyan Li

**Affiliations:** 1 Key Laboratory of Environment and Gene Related Diseases of Ministry Education, Faculty of Public Health, College of Medicine, Xi'an Jiaotong University, Xi'an, Shaanxi, People's Republic of China; 2 Department of Orthopedics Surgery, The Second Affiliated Hospital, College of Medicine, Xi'an Jiaotong University, Xi'an, Shaanxi, People's Republic of China; 3 National Engineering Research Center for Miniaturized Detection Systems, Northwest University, Xi'an, Shaanxi, People's Republic of China; London School of Hygiene and Tropical Medicine, United Kingdom

## Abstract

Kashin-Beck Disease (KBD) is an endemic osteochondropathy, the pathogenesis of which remains unclear now. In this study, we compared gene expression profiles of articular cartilage derived respectively from KBD patients and normal controls. Total RNA were isolated, amplified, labeled and hybridized to Agilent human 1A 22 k whole genome microarray chip. qRT-PCR was conducted to validate our microarray data. We detected 57 up-regulated genes (ratios ≥2.0) and 24 down-regulated genes (ratios ≤0.5) in KBD cartilage. To further identify the key genes involved in the pathogenesis of KBD, Bayesian analysis of variance for microarrays(BAM) software was applied and identified 12 potential key genes with an average ratio 6.64, involved in apoptosis, metabolism, cytokine & growth factor and cytoskeleton & cell movement. Gene Set Enrichment Analysis (GSEA) software was used to identify differently expressed gene ontology categories and pathways. GSEA found that a set of apoptosis, hypoxia and mitochondrial function related gene ontology categories and pathways were significantly up-regulated in KBD compared to normal controls. Based on the results of this study, we suggest that chronic hypoxia-induced mitochondrial damage and apoptosis might play an important role in the pathogenesis of KBD. Our efforts may help to understand the pathogenesis of KBD as well as other osteoarthrosis with similar articular cartilage lesions.

## Introduction

Kashin-Beck Disease (KBD) is an endemic osteochondropathy characterized by chondrocyte necrosis and apoptosis, cartilage degeneration and matrix degradation[1]. KBD usually occurs in children aged 3–12 years with patients exhibiting short stature, joint deformities, and other features caused by impaired epiphyseal growth and ossification[1], [2]. With age, secondary osteoarthritis and deformities of multiple joints become evident in KBD patients[1], [3]. Currently, KBD is mainly prevalent at specific areas of China, Siberia, and North Korea[2], [3]. For instance, more than 2.5 million people suffer from KBD, and about 30 million people are at the risk of KBD in China[4]. Because of osteoarthritis and joint deformities, most of KBD patients without effective treatments will partly or completely lost their abilities to work and even self-care, which not only significantly reduces their quality of life, but also brings heavy medical and financial burdens to society.

Because the pathogenesis of KBD remains unclear, there is not effective approach for preventing and curing KBD now. Most of current treatments of KBD focus on releasing the pain caused by secondary osteoarthritis and joint deformities of KBD[5]. Understanding the pathogenesis of KBD is the key to develop effective measures for preventing and curing KBD. Through epidemiological studies, researchers proposed various environmental etiologic hypotheses for KBD, such as selenium deficiency and cereal contamination by mycotoxins[3], [6], [7], [8], [9]. However, the roles of these environmental risk factors in the pathogenesis of KBD remain under debate now, and none of these environmental etiologic hypotheses can completely explain the pathological changes of KBD. Additionally, recent studies reported that susceptibility genes might contribute to the pathogenesis of KBD[10], [11]. Current epidemiological and genetic study results suggest that KBD was a complex disease, the risk of which was determined by multiple environmental and genetic risk factors[12].

With the rapid development of high-throughput microarray technology, comparing genome-wide gene expression profiles between case and control groups becomes possible now. Significantly differentially expressed genes are likely to involve in the development of target diseases, and may provide insight for pathogenetic studies. Current gene expression analysis can be generally classified into two classes: single gene expression analysis and gene set expression analysis. Single gene expression analysis evaluates the expression level of each individual gene without considering functional relationships among genes. Single gene expression analysis can help us to identify the important genes contributing to the pathogenesis of target diseases. However, since individual gene may have various biological functions, and involve in multiple cellular processes, knowing which gene involved in the development of target diseases is usually inadequate for understanding the pathogenesis of target diseases. To address this issue, gene set expression analysis was proposed[13], [14], and is wildly used in current gene expression studies[15], [16], [17]. Gene set expression analysis attempts to identify differentially expressed gene sets that usually belong to the same gene ontology categories or pathways[13], [14]. Because of combining the information of expression levels and biological functions of multiple genes, gene set expression analysis is believed to be more powerful than traditional single gene expression analysis[13].

To identify the genes, gene ontology categories and pathways involved in the pathogenesis of KBD, we compared genome-wide gene expression profiles of articular cartilage derived respectively from KBD patients and normal controls in this study. Both single gene expression analysis and gene set expression analysis were conducted to ensure the accuracy of our study results, and provide more functional information of differentially expressed genes in KBD vs. control. To the best of our knowledge, this study is the first genome-wide gene set expression analysis of KBD. Our study results may help to unravel the molecular mechanism underlying KBD as well as other osteoarthrosis with similar articular cartilage lesions.

## Results

### Microarray Data Analysis

#### Single Gene Expression Analysis

We detected 57 up-regulated genes (ratios ≥2.0) and 24 down-regulated genes (ratios ≤0.5) in KBD cartilage (detailed in Appendix S1). To further identify the key genes involved in the pathogenesis of KBD, BAM software was applied and identified 12 potential key genes with an average expression ratio 6.64. The 12 genes belong to various functional categories, mainly including apoptosis related BAX (ratio = 3.79), PERP (ratio = 5.59) and TNFRSF11B (ratio = 8.53), metabolism related GALNT1 (ratio = 8.16) and IDH2 (ratio = 4.31), and cytokine & growth factor related TGFBI (ratio = 7.86), IGFBP2 (ratio = 9.70) and IGFBP4 (ratio = 4.13) (Table 1).

**Table 1:** Differently expressed genes identified by BAM.

Gene Symbol	Gene Title	ID	Ratio^a^	P-value
***Apoptosis***				
TNFRSF11B	Tumor necrosis factor receptor superfamily, member 11b (osteoprotegerin)	NM_002546	8.53± 8.01	0.0020
PERP	PERP, TP53 apoptosis effector	NM_022121	5.59±2.38	0.0130
BAX	BCL2-associated X protein	NM_138764	3.79±1.53	0.0546
***Metabolism***				
GALNT1	UDP-N-acetyl-alpha-D-galactosamine:polypeptide N-acetylgalactosaminyltransferase 1 (GalNAc-T1)	XM_031104	8.16±2.62	0.0024
IDH2	Isocitrate dehydrogenase 2 (NADP+), mitochondrial	NM_002168	4.31±0.71	0.0351
***Cytokine & growth factor***				
IGFBP2	Insulin-like growth factor binding protein 2, 36 kDa	NM_000597	9.70±3.73	0.0010
TGFBI	Transforming growth factor, beta-induced, 68 kDa	NM_000358	7.86±3.44	0.0029
IGFBP4	Insulin-like growth factor binding protein 4	NM_001552	4.13±1.53	0.0407
***Cytoskeleton & cell movement***				
TMSL8	Thymosin-like 8	NM_021992	9.65±8.50	0.0011
ACTG1	Actin, gamma 1	NM_001614	6.85±10.68	0.0055
***Miscellaneous***				
GREM1	Gremlin 1, cysteine knot superfamily, homolog (Xenopus laevis)	NM_013372	6.79±4.81	0.0057
DSCR1	Down syndrome critical region gene 1	NM_004414	4.27±3.50	0.0362

a expression ratio is presented as mean± standard error of mean.

#### Gene Set Expression Analysis

Gene ontology expression analysis results are presented in Table 2. Compared with normal controls, we identified 23 up-regulated gene ontology categories as well as 1 down-regulate gene ontology category in KBD. The identified 24 gene ontology categories are grouped based on their biological functions. As shown by Table 2, 9 of 23 up-regulated gene ontology categories involve in the process of apoptosis. The biological functions of the remaining 14 up-regulated gene ontology categories mainly include mitochondrial function (4 gene ontology categories), skeleton development (1 gene ontology category) and extracellular environment (3 gene ontology categories).

**Table 2:** Differently expressed gene ontology categories in KBD vs. control.

Gene Ontology Category	Function	NES^a^	P-value
***Up-regulated***			
APOPTOTIC_PROGRAM	Apoptosis	0.63	<10^−3^
INDUCTION_OF_APOPTOSIS_BY_EXTRACELLULAR_SIGNALS	Apoptosis	0.63	<10^−3^
ANTI_APOPTOSIS	Apoptosis	0.44	<10^−3^
PROGRAMMED_CELL_DEATH	Apoptosis	0.35	0.03
APOPTOSIS_GO	Apoptosis	0.35	0.03
NEGATIVE_REGULATION_OF_APOPTOSIS	Apoptosis	0.41	0.03
NEGATIVE_REGULATION_OF_PROGRAMMED_CELL_DEATH	Apoptosis	0.41	0.03
REGULATION_OF_APOPTOSIS	Apoptosis	0.35	0.05
REGULATION_OF_PROGRAMMED_CELL_DEATH	Apoptosis	0.35	0.05
SKELETAL_DEVELOPMENT	Skeleton development	0.53	0.04
MITOCHONDRIAL_ENVELOPE	Mitochondrion related	0.53	0.03
OXIDOREDUCTASE_ACTIVITY_GO_0016616	Oxidation-reduction reaction related	0.34	0.05
OXIDOREDUCTASE_ACTIVITY__ACTING_ON_CH_OH_GROUP_OF_DONORS	Oxidation-reduction reaction related	0.34	0.05
CELLULAR_RESPIRATION	Aerobic and anaerobic respiration related	0.56	0.03
EXTRACELLULAR_REGION_PART	Extracellular environment	0.39	<10^−3^
EXTRACELLULAR_REGION	Extracellular environment	0.38	<10^−3^
EXTRACELLULAR_SPACE	Extracellular environment	0.35	0.05
PORE_COMPLEX	Membrane passage	0.48	0.03
POSITIVE_REGULATION_OF_HYDROLASE_ACTIVITY	Hydrolysis related	0.47	0.05
PHOSPHORIC_DIESTER_HYDROLASE_ACTIVITY	Phosphodiester hydrolysis related	0.54	0.03
PROTEIN_OLIGOMERIZATION	Protein polymer production	0.57	<10^−3^
COFACTOR_METABOLIC_PROCESS	Cofactor related	0.52	0.04
ANGIOGENESIS	VEGFA related	0.45	0.03
***Down-regulated***			
HYDROLASE_ACTIVITY__HYDROLYZING_O_GLYCOSYL_COMPOUNDS	O-glycosyl hydrolysis	−0.60	<10^−3^

a denotes the normalized enrichment score calculated by GSEA.

Pathway expression analysis identified 149 up-regulated pathways and 20 down-regulated pathways in KBD (detailed in Appendix S2). Based on the biological functions of the differently expressed pathways and previous pathogenetic study results of KBD, 14 important pathways that may contribute to the pathogenesis of KBD are summarized in Table 3. Consistent with our gene ontology expression analysis results, 6 apoptosis-related pathways were significantly up-regulated in KBD, including REACTOME_INTRINSIC_PATHWAY_FOR_APOPTOSIS, FRIDMAN_SENESCENCE_UP, REACTOME_APOPTOSIS, FRIDMAN_IMMORTALIZATION_DN, ALCALA_APOPTOSIS and BIOCARTA_CERAMIDE_PATHWAY. We also found that BIOCARTA_AT1R_PATHWAY that involved in the activation of apoptosis-related JNK pathway[18], appeared to significantly up-regulate in KBD. Another important finding of our pathway expression analysis is that 5 hypoxia-related pathways were significantly up-regulated in KBD, including HARRIS_HYPOXIA, SEMENZA_HIF1_TARGETS, WINTER_HYPOXIA_METAGENE, ELVIDGE_HYPOXIA_BY_DMOG_UP and LEONARD_HYPOXIA. Additionally, KEGG_PEROXISOME playing an important role in the detoxification of reactive oxygen species (ROS), was significantly up-regulated in KBD.

**Table 3:** Selected pathways significantly up-regulated in KBD vs. control.

Pathway	Description	NES^a^	P-value
REACTOME_INTRINSIC_PATHWAY_FOR_APOPTOSIS	Genes involved in intrinsic pathway for apoptosis	0.62	<10^−3^
FRIDMAN_SENESCENCE_UP	Genes up-regulated in senescent cells.	0.54	<10^−3^
REACTOME_APOPTOSIS	Genes involved in apoptosis	0.42	<10^−3^
FRIDMAN_IMMORTALIZATION_DN	Genes down-regulated in immortalized cell lines.	0.64	<10^−3^
ALCALA_APOPTOSIS	Genes able to induce cell death in an expression cDNA library screen.	0.45	<10^−3^
BIOCARTA_CERAMIDE_PATHWAY	Ceramide signaling pathway	0.67	<10^−3^
HARRIS_HYPOXIA	Genes known to be induced by hypoxia	0.62	<10^−3^
SEMENZA_HIF1_TARGETS	Genes that are transcriptionally regulated by HIF1A	0.77	<10^−3^
WINTER_HYPOXIA_METAGENE	Genes regulated by hypoxia, based on literature searches.	0.51	<10^−3^
ELVIDGE_HYPOXIA_BY_DMOG_UP	Genes up-regulated in MCF7 cells (breast cancer) treated with hypoxia mimetic DMOG	0.59	0.027
LEONARD_HYPOXIA	Genes up-regulated in HK-2 cells kidney tubular epithelium under hypoxia and down-regulated on re-oxygenation	0.59	<10^−3^
KEGG_PEROXISOME	Peroxisome	0.49	<10^−3^
GALLUZZI_PERMEABILIZE_MITOCHONDRIA	Proteins that permeabilize mitochondria.	0.48	<10^−3^
BIOCARTA_AT1R_PATHWAY	Angiotensin II mediated activation of JNK pathway via Pyk2 dependent signaling	0.59	<10^−3^

a denotes the normalized enrichment score calculated by GSEA.

### qRT-PCR Validation of Microarray data

**Figure 1:**
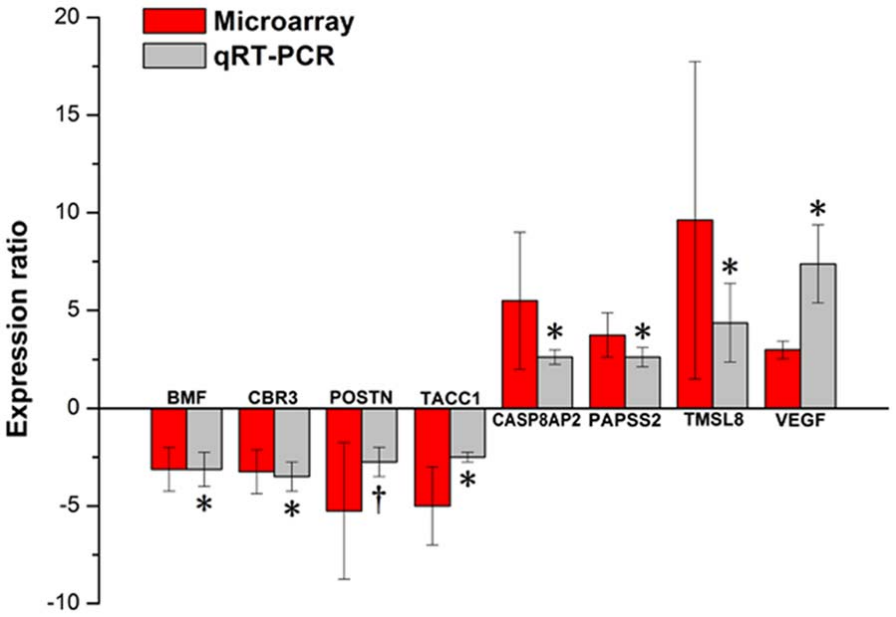
Histogram showing the expression values of the selected 8 genes measured by microarray and qRT-PCR. * indicates p<0.05 and † indicates p<0.01, calculated by paired t test.

4 up-regulated (ratios >2.0) and 4 down-regulated genes (ratios <0.5) were selected for quantitative real-time reverse transcription polymerase chain reaction (qRT-PCR) to validate our microarray data. qRT-PCR results are presented in Figure 1. We observed increasing expression levels of CASP8AP2, PAPSS2, TMSL8 and VEGF in KBD compared to normal controls (ratios >2.0). The expression levels of BMF, CBR3, POSTN and TACC1 appeared to decrease in KBD than in normal controls (ratios <0.5). Gene expression patterns of the 8 genes in qRT-PCR are consistent with that in microarray experiment, confirming the validity of our microarray data.

## Discussion

With high-throughput microarray technology, we compared gene expression profiles of cartilage derived from KBD patients and normal controls, respectively. Our study results suggest that chronic hypoxia-induced mitochondrial damage and apoptosis might play an important role in the pathogenesis of KBD. In the following, we present some of the identified differently expressed genes and gene sets, which may contribute to the pathogenesis of KBD.

### Apoptosis

We found that apoptosis-related genes and gene sets were up-regulated in KBD. Among the 12 potential key genes, 3 genes involve in the process of apoptosis, including BAX, PERP and TNFRSF11B. TNFRSF11B[19] and BAX[20] have been reported to be associated with osteoarthrosis by previous studies. Additionally, 9 apoptosis-related gene ontology categories and 6 apoptosis-related pathways were significantly up-regulated in KBD compared to normal controls, which supports the importance of apoptosis in the pathogenesis of KBD. Our results are consistent with the fact that abnormal chondrocyte apoptosis is one of the primary pathological changes of KBD[1], [21]. However, the molecular mechanism underlying abnormal chondrocyte apoptosis in KBD remains elusive.

### Hypoxia

An important finding of this study is that 5 hypoxia-related pathways were significantly up-regulated in KBD, suggesting that hypoxia might contribute to the pathogenesis of KBD. This result is consistent with that of previous studies, which found that hypoxia might play an important role in the pathogenesis of osteoarthritis and rheumatoid arthritis[22], [23], [24]. The molecular mechanism of hypoxia-induced articular lesions may partly be explained by increased production of ROS under hypoxia[25], [26], which are able to promote lipid peroxidation, protein oxidation, and induce mitochondrial damage and apoptosis[27], [28]. In vivo, ROS are mainly detoxified at peroxisome through special enzymes, such as glutathione peroxidases, superoxide dismutases and catalases. In this study, KEGG_PEROXISOME pathway playing a key role in the detoxification of ROS, was significantly up-regulated in KBD, which suggested the increased activity of ROS metabolism in KBD patients compared to normal controls.

### Mitochondria related

It is well documented that mitochondria play a critical role in apoptosis[29]. Several mitochondria-related proteins that can activate intrinsic apoptotic programs, have been identified, such as Bcl-2 and BAX [30]. In this study, we found that BAX was up-regulated in KBD, which was also observed by previous study of KBD[20]. BAX is one of the proapoptotic members of apoptosis regulator proteins Bcl-2 family. It has been demonstrated that BAX protein laying on the surface of mitochondria, is able to damage mitochondria and induce apoptosis through permeabilizing mitochondrial membrane[31], [32]. We also found that BAX involved GALLUZZI_PERMEABILIZE_MITOCHONDRIA pathway that could permeabilize mitochondria membrane during mitochondria-mediated apoptosis, was significantly up-regulated in KBD. Although we observed a marginal significant p value of 0.0546 for BAX in our single gene expression analysis, previous and our study results suggest that mitochondrial BAX-induced apoptosis might contribute to the pathogenesis of KBD. Further studies are needed to investigate the potential effect of BAX on the risk of KBD.

It has been suggested that hypoxia played an important role in the pathogenesis of osteoarthrosis, such as osteoarthritis and rheumatoid arthritis [22], [23], [24]. However, the potential molecular mechanism underlying hypoxia-induced articular lesions in osteoarthrosis remains under debate. Researchers proposed various hypothesis about the role of hypoxia in the pathogenesis of osteoarthrosis, such as abnormal energy metabolism and immune & inflammation[33]. Our previous study observed abnormal functional changes of mitochondria in the cartilage of KBD, and suggested that mitochondrial dysfunction might contribute to the abnormal chondrocyte apoptosis of KBD [34]. Based on our study results, we suggest that hypoxia-induced mitochondrial damage and apoptosis might play an important role in the pathogenesis of KBD. Previous study results may provide some evidence for this etiologic hypothesis. It has been reported that hypoxia could increase mitochondrial production of ROS[25], [26], which were able to induce apoptosis through activating mitochondrial JNK pathway[35]. Another study found that activated JNK pathway could promote the phosphorylation of BAX protein following by triggering mitochondria-mediated apoptosis[36]. In this study, we found that BAX gene, 6 apoptosis related pathways, 5 hypoxia-related pathways, ROS metabolism–related KEGG_PEROXISOME pathway, and BIOCARTA_AT1R_PATHWAY pathway involved in the activation of JNK pathway, were simultaneouly up-regulated in KBD, which supported the role of hypoxia-induced mitochondrial damage and apoptosis in the pathogenesis of KBD.

Mycotoxins, selenium deficiency and humic substance are regarded as three important environmental risk factors of KBD[1], [3], [6], [37], [38]. The molecular mechanisms of cartilage lesions caused by mycotoxins, selenium deficiency and humic substance remain unclear now. Our study results may provide some evidence for understanding the roles of mycotoxins, selenium deficiency and humic substance in the pathogenesis of KBD. Previous studies have reported that mycotoxins could result in hypoxia[39], and increase the production of ROS in vivo[40]. In this study, we observed that hypoxia and ROS metabolism related pathways were significantly up-regulated in KBD. Based on the biological effects of mycotoxins and our study results, we may infer that the causative effects of mycotoxins might partly be attributed to hypoxia and ROS induced cartilage lesions in KBD. Additionally, as an important metabolism enzyme of ROS, glutathione peroxidase is a selenium-containing enzyme, and its protective effect against ROS largely depends on the presence of selenium[41]. Selenium deficiency may result in the loss of protective function of glutathione peroxidase against ROS, and therefore increase susceptibility to KBD[12]. For humic substance, it was suggested that humic substance could induce oxidative stress (imbalance between the production and detoxification of ROS), which might contribute to the cartilage lesions of KBD [42], [43], [44]. The significant up-regulation of ROS metabolism–related pathway in KBD observed by our study may provide some evidence for this etiologic hypothesis.

Two aspects of our study deserve further emphasizing. First, because KBD usually becomes evident in children coming from specific areas with similar environment (for example selenium deficiency), the causative factors of KBD should be less than that of other osteoarthrosis, such as osteoarthritis and rheumatoid arthritis, which occur in adults and mostly in elderly persons. The etiologic heterogeneity of KBD should be smaller than that of other osteoarthrosis, the risks of which are determined by cumulative effects of multiple genetic and environmental causative factors during life span. Therefore, KBD is ideal for molecular mechanism studies of articular cartilage lesions in osteoarthrosis. The differently expressed genes and gene sets identified by this study may provide insight for addressing the pathogenesis of KBD as well as other osteoarthrosis with similar articular cartilage lesions. Second, gene set expression analysis is first applied to the pathogenetic studies of KBD in this study. Because of combining the functional and expressing information of multiple genes, gene set expression analysis should be more powerful than traditional single gene expression analysis. It may help us to identify important pathways involved in the pathogenesis of KBD, which are usually difficult to be detected by single gene expression analysis.

In summary, we compared genome-wide gene expression profiles of KBD and normal controls, and identified a set of differently expressed genes, gene ontology categories and pathways. Based on the results of this study, we suggest that chronic hypoxia induced mitochondrial damage and apoptosis might play an important role in the pathogenesis of KBD. Our efforts may help to understand the molecular mechanism underlying KBD as well as other osteoarthrosis with similar articular cartilage lesions.

## Materials and Methods

All studies were approved by the Institutional Review Board of Xi'an Jiaotong University. Informed-consent documents were written by all KBD patients and the relatives of donors before entering this study.

### Cartilage Sample Collection

**Figure 2:**
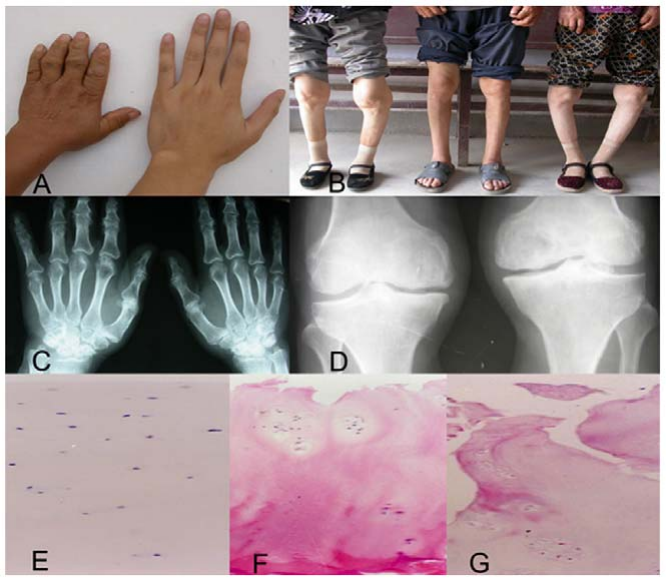
Characteristics of Kashin-Beck disease (KBD). A&B, images of left hand(A, left male patient aged 55 years) and knees (B, left female patient aged 44 years, middle male patient aged 42 years, right female patient aged 49 years) from representative patients with grade II or III KBD; C, radiographic images of left and right hands of a KBD patient exhibiting shortened phalanges, enlarged bone ends of phalanges, and narrowed joint space; D, radiographic images of left and right knees of a KBD patient exhibiting widened and narrowed joint space; E–G, hematoxylin and eosin staining of adult articular cartilage from a healthy subject (E) and two patients with grade II (F) or III (G) KBD (original magnification×100).

Articular cartilage samples were collected from 9 KBD patients and 9 normal controls, respectively. All study subjects were Chinese Han. Every year, there are 100 randomly selected patients with serious KBD undergoing free total knee replacement surgery, which was supported by Shaanxi province of China. Our 9 KBD patients were randomly selected from the KBD patients undergoing free total knee replacement surgery, and came from the KBD prevalent areas-Linyou county and Yongshou county of Xi'an city of Shaanxi province of China. Based on the radiography of right hand, knee and hip joints, and cartilage sections after hematoxylin and eosin (H&E) staining (Figure 2), KBD patients were diagnosed as having grade II or III KBD according to the KBD clinical diagnosis criteria of China (Diagnostic code: GB16395-1996)[45]. The cartilage samples of normal controls were collected from the knees of fresh cadaver within 8 hours of death caused by traffic accidents. 9 cadaver donors came from the non-KBD prevalent counties of Xi'an city, the economic conditions and living habits of which were similar to that of Linyou and Yongshou counties. KBD, genetic bone and cartilage diseases, osteoarthritis and rheumatoid arthritis were excluded for the 9 cadaver donors by cartilage section examination with H&E staining. All cartilage samples were derived from the same anatomic area of femoral condyles of knee. The obtained cartilage samples were rapidly dissected and frozen in liquid nitrogen, and subsequently stored at −80°C until RNA extraction. Specific for this study, four and five KBD-control pairs, matched for age and sex, were used for microarray and qRT-PCR, respectively (Table 4).

**Table 4:** KBD-control sample pairs used for microarray and qRT-PCR.

	KBD		Control	
	Age(years)	Sex	Age(years)	Sex
Microarray sample set	55	Male	55	Male
	52	Male	54	Male
	49	Female	48	Female
	42	Male	37	Male
qRT-PCR sample set	69	Male	60	Male
	57	Female	58	Female
	51	Male	58	Male
	50	Male	56	Male
	44	Female	34	Female

### RNA Preparation

Frozen cartilage of each sample was first rapidly ground in liquid nitrogen using freezer mill (SPEX CertiPrep, Metuchen, NJ, USA). Total RNA were then isolated from cartilage samples using the Agilent Total RNA Isolation Mini kit (Agilent Technologies, Santa Clara, CA, USA) following manufacturer recommended protocol. The quality and integrity of isolated total RNA were evaluated with 1% agarose gel electrophoresis.

### Microarray Hybridization

Total RNA were first transcribed into cDNA, and labeled with CyDye using the Amino Allyl MessageAmp aRNA Kit (Applied Biosystems, Austin, TX, USA). For each KBD-control pair, 0.5 µg of labeled cDNA were then purified separately and mixed together with hybridization buffer (Agilent In Situ Hybridization Plus kit). Agilent Human 1A 22 k Whole Genome microarray (G4110B) that contains 22,575 oligonucleotides probes representing 21,073 human genes, was applied for microarray hybridization following Agilent recommended protocol. Hybridization signals were recorded by Agilent G52565BA scanner, and analyzed by Feature Extraction 9.3 (Agilent Technologies) and Spotfire 8.0 (Spotfire Inc., Cambridge, MA, USA) software. The quality of fluorescent spots was evaluated by Feature Extraction 9.3. The fluorescent spots that failed to pass the quality control procedure were excluded from further analysis. Linear and LOWESS normalization were conducted to eliminate possible dye-related bias in our microarray data. The gene expression data obtained from Spotfire 8.0 was imported into Excel spreadsheets (Microsoft Corp., Redmond, WA, USA) for downstream data analysis. Our microarray data was MIAME compliant and has been deposited in the MIAME compliant database ArrayExpress (Accession number:E-MEXP-3196).

### Microarray Data Analysis

#### Single Gene Expression Analysis

Gene expression ratios were calculated from the microarray data. Up-regulated and down-regulated genes were identified by ratios ≥2.0 and ≤0.5, respectively. To further identify the key genes involved in the pathogenesis of KBD, Bayesian analysis of variance for microarrays (BAM, http://www.bamarray.com/) software was applied to our genome-wide microarray data [46,47]. The overlapped genes between the gene set identified by BAM and the gene set with ratios ≥2.0 or ≤0.5 were regarded as the potential key genes of KBD in this study. BAM was running under developers recommended parameters. Statistical p values were calculated for each gene using Agilent p value log ratio algorithm (Agilent Technologies)[1], defined by



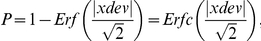



where Erf was calculated by



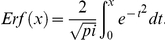



Erf(x) denotes twice the integral of Gaussian distribution with mean value 0 and variance 0.5; xdev denotes the deviation of log ratio from 0.

#### Gene Set Expression Analysis

To investigate the gene expression patterns of KBD in the context of molecular functions, biological processes and cellular components, Gene Set Enrichment Analysis (GSEA, http://www.broadinstitute.org/gsea/index.jsp) software was used here to evaluate the expression levels of gene sets, which were predefined according to known biological knowledge, such as gene ontology categories and biochemical pathways[13], [14]. During gene set expression analysis, GSEA calculated an enrichment score (ES) for each gene set, which reflected the over-represented degree of corresponding gene set. The gene sets with extreme ES values were suggested to be correlated with study phenotypes by GSEA[13], [14]. GSEA gene ontology collection 3.0 and curated gene set collection 3.0 were downloaded from the Molecular Signature Database (http://www.broadinstitute.org/gsea/msigdb/index.jsp), and applied to GSEA for gene ontology and pathway expression analysis, respectively. GSEA gene ontology collection 3.0 contains 1,454 gene ontology categories. GSEA curated gene set collection 3.0 contains 3,272 molecular pathways. Statistical p values were calculated by GSEA for each gene set. Significant threshold was set as p values ≤0.05 in this study.

### qRT-PCR Validation

qRT-PCR was conducted to validate our microarray data using an independent sample set (Table 4). 4 up-regulated genes (ratios >2.0) and 4 down-regulated genes (ratios <0.5) in our microarray experiment were randomly selected for qRT-PCR, including BMF, CBR3, CASP8AP2, PAPSS2, POSTN, TACC1, TMSL8 and VEGF. Total RNA were isolated from cartilage samples, and prepared in the same way as used by the microarray experiment. The isolated total RNA were converted into cDNA using Superscript II reverse transcriptase (Invitrogen, Carlsbad, CA, USA). Amplification and detection of cDNA were performed using ABI 7500 Real-Time PCR Detection System (Applied Biosystems) following manufacturer recommended protocol. Glyceraldehyde-3-phosphate dehydrogenase (GAPDH) was simultaneously assayed by qRT-PCR as an endogenous invariant control for data normalization. All primer and probe sets of qRT-PCR were supplied by TaqMan Gene Expression Assays (Applied Biosystems). The expression values of the 8 genes were normalized to the amount of GAPDH, and used for calculating expression ratios. Paired t test was conducted to assess the significance levels of expression differences of the 8 genes between KBD patients and normal controls.

## Supporting Information

Appendix S1 Single gene expression analysis results in KBD vs. control.
(XLS)

Appendix S2 Pathway expression analysis results in KBD vs. control.
(XLS)
